# From fertilizer to insecticide: urban leaf litter chemistry alters the survival landscape of *Aedes aegypti*

**DOI:** 10.1002/ps.70466

**Published:** 2026-01-02

**Authors:** Ana Luiza Caldatto, Gilberto Dinis Cozzer, Heloise Restello Remus, Raquel de Brito, Monica Santin Zanatta Schindler, Cássia Alves Lima‐Rezende, Jacir Dal Magro, Renan de Souza Rezende

**Affiliations:** ^1^ Communitarian University of Chapecó Region – Unochapecó Chapecó Santa Catarina Brazil

**Keywords:** allelopathy, phenotypic plasticity, urban ecology, integrated pest management, secondary metabolites

## Abstract

**BACKGROUND:**

Urban leaf litter accumulating in water‐filled containers may function as either a resource or a stressor for *Aedes aegypti* larvae, yet the chemical and botanical drivers of these contrasting effects remain poorly understood. We combined untargeted metabolite profiling with factorial life‐history bioassays to examine how leachates from two dominant street trees, *Tipuana tipu* and *Handroanthus heptaphyllus*, influence mosquito life story. First‐instar larvae were exposed to 25%, 50% or 100% leachate aged 7 or 14 days.

**RESULTS:**

The *T. tipu* leachates were defined by persistent oxalic acid (cyclohexyl hexyl ester) and condensed tannins over 14 days of decay, whereas *H. heptaphyllus* rapidly lost most phenolics within the first week, shifting to profiles dominated by short‐chain alkenes. At 25% dilution, *T. tipu* reduced mortality to 7% and produced adults with greater wing lengths than controls. However, mortality was >90% in the 50% and 100% *T. tipu* treatments, independent of leachate age. By contrast, *H. heptaphyllus* never produced mortality > 16% across all concentration–age combinations. Adult body size responded nonlinearly, with 100% *T. tipu* aged 14 days generated the smallest adults, whereas the largest adults emerged from 25% *T. tipu*.

**CONCLUSION:**

These results indicate that *T. tipu* can shift from a nutritional subsidy to a potent chemical stressor depending on concentration and aging, whereas *H. heptaphyllus* exerts consistently mild effects. Urban leaf litter therefore represents an overlooked but influential driver of mosquito performance in city environments. © 2026 The Author(s). *Pest Management Science* published by John Wiley & Sons Ltd on behalf of Society of Chemical Industry.

## Graphical abstract

Chemical profiling and bioassays reveal that leaf‐litter leachates from urban trees flip between nourishing and killing *Aedes aegypti* larvae: dilute *Tipuana tipu* boosts growth, whereas concentrated, aged extracts are >90% lethal. Species‐specific chemistry thus turns street trees into potential self‐renewing tools for integrated vector control.

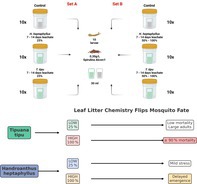

## 1 INTRODUCTION

Arboviral diseases vectored by *Aedes (Stegomyia) aegypti* Linnaeus, 1762 continue to intensify as global public health threats, driven in large part by the species' remarkable ecological alignment with urban environments.^1^ Originally endemic to sub‐Saharan Africa, *A. aegypti* has evolved to exploit the anthropogenic matrix of water‐filled receptacles and high‐density human populations that define contemporary cities.^1^, ^2^ Structural inequities, including chronic sanitation deficits and pervasive socio‐economic vulnerability, expand this ecological niche by sustaining litter capable of retaining water and constraining the operational reach of vector control programs, thereby amplifying urban infestation.^1^, ^2^, ^3^ Females oviposit desiccation‐resistant eggs just above the waterline; subsequent inundation cues synchronous larval emergence.^3^, ^4^

Once submerged, *A. aegypti* larvae rely primarily on microbial biofilms and particulate organic matter as nutritional substrates.^4^, ^5^ These resources, however, are inherently limited, imposing strong density‐dependent constraints that decelerate growth^6^, extend developmental timelines and increase larval mortality.^7^ Nutritional quality during larval stages emerges as a central driver of adult phenotype: cohorts with abundant resources yield larger, longer‐lived and more fecund adults,^8^ whereas dietary restriction may elevates fluctuating asymmetry, an established proxy for developmental instability.^9^ Environmental factors such as temperature, larval crowding and chemical signaling further modulate these dynamics, yet the mechanistic intersections among these cues remain incompletely understood.^10^, ^11^, ^12^

Chemical insecticides remain central to *A. aegypti* control strategies, yet their sustained application has accelerated the evolution of both metabolic and target‐site resistance, while concurrently raising ecological and human health concerns.^13^, ^14^, ^15^ By contrast, plant‐derived secondary metabolites present a promising, biologically grounded alternative.^9^, ^16^, ^17^ These compounds may act across multiple physiological pathways, exerting diffuse and nonspecific selective pressures that reduce the likelihood of resistance development.^15^, ^16^ Additionally, they are typically characterized by low vertebrate toxicity and rapid environmental degradation.^18^ Among these, leaf‐litter leachates are particularly compelling:^9^, ^19^ as senescent foliage undergoes microbial decomposition, ^20^, ^21^ hydrophilic and semi‐polar allelochemicals leach into the water column, generating a chemically complex milieu capable of eliciting both lethal and sublethal effects in mosquito larvae.^9^, ^19^

Litter inputs into urban containers are shaped by local street‐tree composition. In Chapecó, Santa Catarina (Brazil), the Urban Greening Plan documents a striking dominance of *Tipuana tipu* (Fabaceae; 23% of street trees) and *Handroanthus heptaphyllus* (Bignoniaceae; 13%).^22^ *T. tipu* foliage is notably rich in tannins and produces extracts with demonstrated antibacterial and antioxidant activity,^23^ whereas bark and wood of *H. heptaphyllus* exhibit a range of bioactivities, including antibacterial, anti‐inflammatory and antitumor effects.^24^ Despite these well‐characterized pharmacological properties, the larvicidal potential of their foliar leachates against *A. aegypti* remains unquantified. Given their prevalence in the urban landscape, these species represent a continuous, embedded source of bioactive detritus with untapped potential for integrative vector control.

The fast‐growing leguminous tree *T. tipu* (Benth.) O. Kuntze (Fabaceae), native to northern Argentina and southern Bolivia, is widely cultivated along urban thoroughfares in southern and southeastern Brazil owing to its expansive canopy and ornamental appeal. Beyond its landscaping value, *T. tipu* has a well‐documented ethnobotanical history, traditionally employed as a laxative and astringent, uses largely attributed to its elevated tannin content.^23^ *H. heptaphyllus* (Vell.) Mattos (Bignoniaceae), commonly known as purple ipê, occurs across Bolivia, Paraguay and at least 10 Brazilian states, including Santa Catarina. The species is widely recognized in traditional medicine for treating gastrointestinal ailments, and pharmacological studies have corroborated its antibacterial, anti‐inflammatory and antitumor properties, particularly in bark and wood extracts.^24^

Here, we test the hypothesis that leaf‐litter leachates from *T. tipu* and *H. heptaphyllus* modulate *A. aegypti* life‐history traits in a species‐, concentration‐ and time‐dependent manner, reflecting dynamic shifts in chemical composition during leaf litter decomposition. We specifically predict that variation in leaching duration and solute concentration will differentially, in a context‐dependent, influence larval survival, developmental timing, adult body size and wing symmetry. To address this, our objective was to: (i) characterize the metabolite profiles of *T. tipu* and *H. heptaphyllus* leachates across defined leaching intervals and concentrations, and (ii) quantify their impacts on mortality, ontogenetic progression and morphological traits in *A. aegypti*. By directly linking species‐specific litter chemistry to mosquito fitness outcomes, our findings aim to inform urban landscape design and identify context‐dependent botanical resources for integration into ecologically grounded vector control strategies.

## 2 MATERIAL AND METHODS

### 2.1 Study site and insect colony

All experiments were conducted under controlled climatic conditions (27 ± 2 °C, 70–80% relative humidity (RH), 12 h:12 h, light: dark photoperiod) in the Ecological Entomology Laboratory (LABENT‐Eco) at the Community University of the Chapecó Region (Unochapecó), Chapecó, Santa Catarina, Brazil (27° 06′ S, 52° 37′ W; Köppen‐Geiger classification: Cfa). The *A. aegypti* colony was established in 2018 and is refreshed annually with field‐collected adults to maintain genetic heterogeneity; no artificial selection is imposed. Eggs were stimulated to hatch by submerging oviposition papers in 1 L water contained in 2‐L glass beakers. First‐instar larvae were collected within 24 h postimmersion; diapause occurrence was not quantified. Stock cultures were maintained in plastic trays (40 × 30 × 6 cm) containing 500–600 larvae and held under identical abiotic conditions throughout the experimental period.

### 2.2 Plant material and leachate preparation

Tree inventory data were obtained from the municipal ‘Plano de Arborização Urbana de Chapecó’^22^ which conducted a street‐tree census of the urban zones in Chapecó in July 2019 (*c*. 4853 trees). Senescent leaves of *T. tipu* (Fabaceae) and *H. heptaphyllus* (Bignoniaceae) were collected in June 2024 from two urban locations in Chapecó, Brazil (*T. tipu*: General Osório Avenue; *H. heptaphyllus*: Palmitos Street). Voucher specimens were deposited in the UNO Herbarium under accession nos UNO‐Tt24 and UNO‐Hh24. Collected leaves were rinsed with distilled water to remove surface litter, air‐dried at 25 °C for constant weight. For each species, 30 g leaf litter were immersed in 1 L distilled water within sealed 2‐L Erlenmeyer flasks and incubated at 20 °C under a 16 h:8 h, light: dark photoperiod for either 7 or 14 days without agitation (protocol modified from Beleza *et al*., 2019). Leachates were subsequently filtered through Whatman No. 1 paper and refrigerated at 4 °C for ≤24 h before experimental use.

### 2.3 Experimental design

**Figure 1:**
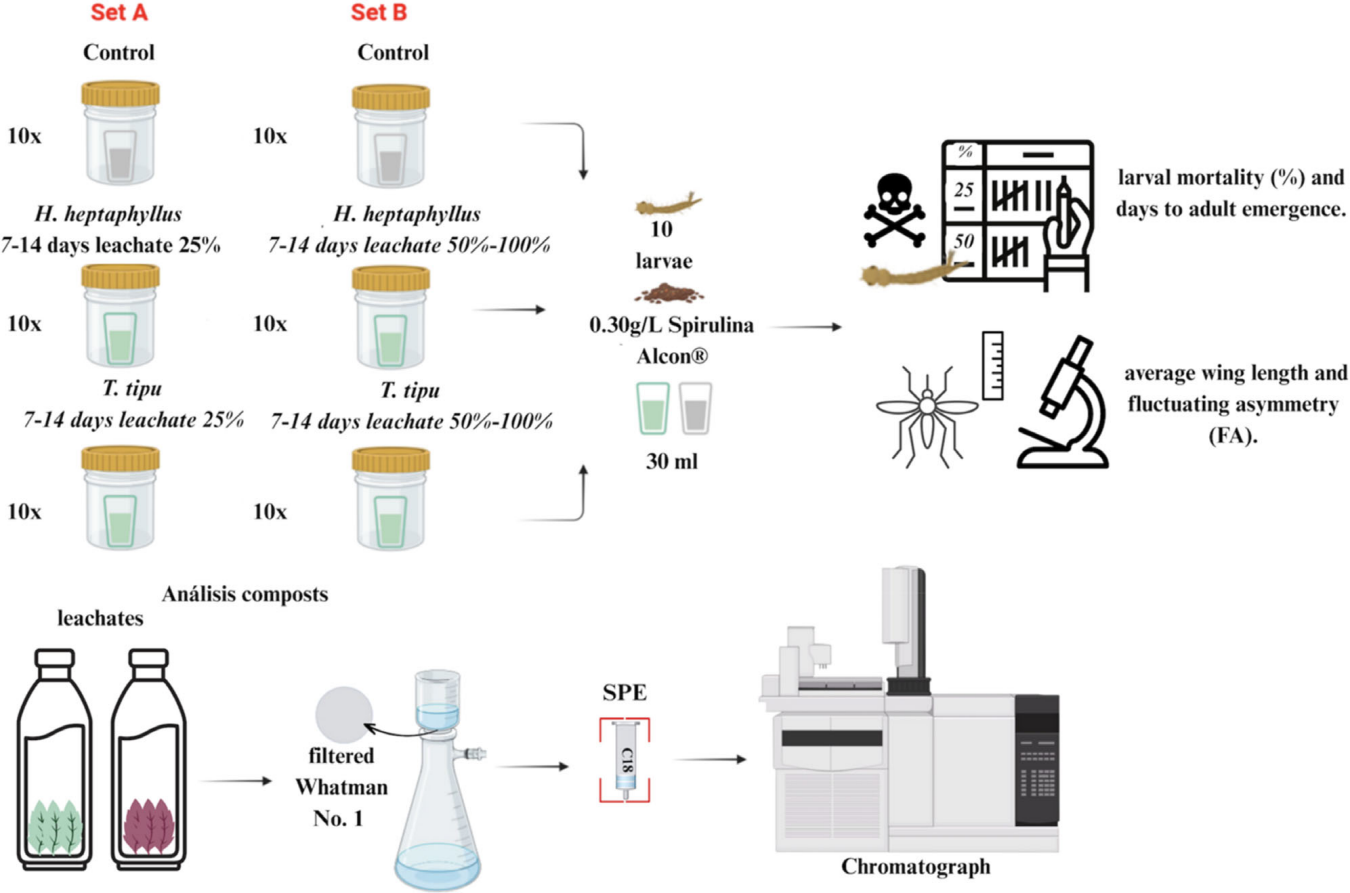
Experimental design of microcosm assays testing the interactive effects of litter species, leaching duration and leachate concentration on *A. aegypti* life‐history traits.

Experimental treatments were organized into two independent sets. Set A comprised five treatments with 10 replicates each (*n* = 50): (i) control with distilled water and standard larval food only; (ii) *T. tipu* leachate aged 7 days (D7) at 25% concentration; (iii) D14 *T. tipu* leachate at 25%; (iv) D7 *H. heptaphyllus* leachate at 25%; and (v) D14 *H. heptaphyllus* leachate at 25% [Fig. 1(a)]. Set B comprised nine treatments, also with 10 replicates each (*n* = 90): (i) control; (ii) D7 *T. tipu* leachate at 50%; (iii) D7 *T. tipu* leachate at 100%; (iv) D14 *T. tipu* leachate at 50%; (v) D14 *T. tipu* at 100%; (vi) D7 *H. heptaphyllus* leachate at 50%; (vii) D7 *H. heptaphyllus* leachate at 100%; (viii) D14 *H. heptaphyllus* leachate at 50%; and (ix) D14 *H. heptaphyllus* leachate at 100% [Fig. 1(b)]. Each replicate consisted of 10 1^st^‐instar *A. aegypti* larvae introduced into 30 mL treatment solution, yielding a density of 0.33 larvae mL^−1^.

### 2.4 Microcosm medium and larval husbandry

A basal diet solution was freshly prepared for each trial by dissolving 0.30 g L^−1^ of *Spirulina* powder (Alcon, Camboriú, Brazil) in distilled water,^7^ weighed on a precision analytical balance (model SKU‐M, 0.0001 g resolution; Bel Engineering, Monza, Italy) and homogenized by stirring for 3 min. Treatment media were prepared by replacing the corresponding volume of distilled water with leaf leachate (25%, 50% or 100% v/v) and adding the diet solution to a final volume of 30 mL. *Spirulina* was added at the same final concentration to all treatments to maintain a common baseline food level.^7^ This approach prevents starvation effects and ensures that differences across treatments reflect leaf‐litter chemistry and its interactions with microbial dynamics, rather than variation in nutritional availability *per se*.

Microcosms were inspected daily. Dead larvae and pupae were removed and counted, and pupal exuviae were retained to estimate developmental timing. Upon emergence, adults were aspirated into mesh‐covered holding cups, chilled at 4 °C for 2 min, and sexed under a stereomicroscope (Stemi 508, ×40 magnification; Zeiss, Jena, Germany). Both wings were detached, mounted in Hoyer's medium, and measured from the proximal notch to the distal tip using an ocular micrometer (0.01‐mm precision). Mean wing length was used as a proxy for adult body size, while fluctuating asymmetry (FA) was calculated as the absolute difference between left and right wing lengths (|L – R|).

### 2.5 Chemical characterization of leachates

For the extraction of compounds present in the leachate of *H. heptaphyllus* and *T. tipu* 7 and 14 days, the solid phase extraction (SPE) technique, eluted with 5 mL f methanol, was employed using Chromabond C18 cartridges (500 mg, 6 mL; machery‐Nagel, Düren, Germany) in the 12‐position ROTI®XBond system, coupled to a KNF N 816.3, N816.3KT diaphragm vacuum pump. During this process, the compounds were adsorbed onto the cartridges, whereas the aqueous phase was discarded. The adsorbed compounds were then eluted with 5 mL methanol to ensure complete transfer of the analytes from the cartridge to the eluent. As a result, the analytes extracted from the water samples were pre‐concentrated 200‐fold to increase their detection. The eluted samples were placed in 1.5‐mL vials, labeled, and subjected to gas chromatography‐mass spectrometry (GC–MS) analysis.

Volatile and semi‐volatile constituents of leaf leachates were analyzed using an 7890B gas chromatograph coupled to a 5977A quadrupole mass spectrometer (Agilent Technologies, Santa Clara, CA, USA), equipped with an HP‐5 MS capillary column (30 m × 0.25 mm × 0.25 μm). Helium was used as the carrier gas at a flow rate of 1.2 mL min^−1^. The oven temperature was programmed as follows: initial hold at 40 °C for 2 min, ramped to 200 °C at 5 °C min^−1^, then to 300 °C at 10 °C min^−1^, with a final hold of 3 min. The ion source temperature was set at 230 °C, transfer line at 250 °C, and electron ionization was performed at 70 eV, scanning a mass range of 40–400 *m/z*. Compound identifications are based on spectral matching (≥80%) against the NIST 17 library and should be interpreted as provisional annotations, not definitive structural identifications. Total phenolic content and condensed tannins were quantified colorimetrically using a 96‐well UV‐visible microplate reader. Phenolics were assessed via the Folin–Ciocalteu assay,^25^ and tannins via the vanillin‐HCl method.

### 2.6 Statistical analyses

All statistical analyses were performed in R v4.1.3. For Set A, two‐way generalized linear mixed‐effects models (GLMMs) were fitted using the *glmer* function (lme4 v1.1–32), with fixed effects for species (*T. tipu*, *H. heptaphyllus*, control) and leaching time (7 *versus* 14 days).^26^ Set B was analyzed using three‐way GLMMs, incorporating leachate concentration (50% *versus* 100%) as an additional fixed factor. Response variables included larval mortality (%), days to adult emergence, mean wing length and fluctuating asymmetry (FA). Random intercepts for microcosm ID and sex accounted for nonindependence of repeated measures within containers.^26^

Error distributions were selected based on diagnostic half‐normal plots (hnp package),^27^ and quasi‐likelihood corrections were applied when overdispersion or underdispersion was detected. Gaussian error structures were used for mortality, development time and wing length, whereas FA was modeled with a Poisson distribution. Significance of fixed effects was evaluated via likelihood ratio tests comparing full and nested models (χ^2^). *Post hoc* pairwise comparisons were adjusted using Tukey's honestly significant difference (HSD) implemented in the emmeans package.^26^ Residual diagnostics confirmed assumptions of homoscedasticity and normality where applicable.

## 3 RESULTS

### 3.1 Chemical fingerprint of leaf‐litter leachates

The GC–MS analysis resolved 26 compounds exceeding 2% of the total ion chromatogram in at least one of the four leachate types. Of these, 10 compounds were unique to *H. heptaphyllus* and 14 (days of leaf leaching) to *T. tipu* (Table 1). In *H. heptaphyllus*, the D7 leachate (7 days of leaf leaching) was dominated by branched‐chain alkenes, particularly 2‐pentene, 2,3‐dimethyl‐ (9.79%) and 1‐pentene, 3‐ethyl‐3‐methyl‐ (8.93%). By D14 (14 days of leaf leaching), these volatiles had largely been supplanted by higher molecular weight compounds, including 2‐pentene, 4,4‐dimethyl‐ (14.47%) and the methoxy ester of 4‐pentenoic acid (8.32%), suggesting a temporal shift from short‐chain hydrocarbons to more oxidized derivatives.

**Table 1:** Area (%) of tentatively annotated volatile and semi‐volatile compounds, retention times (RT) and Kovats retention indices (RI) detected in D7 and D14 leachates of *H. heptaphyllus* and *T. tipu* by GC/MS.

Compound	RT	RI Kovats	*H. heptaphyllus*		*T. tipu*	
			D7	D14	D7	D14
2‐Pentene, 2,3‐dimethyl‐	3154	800.5	9.79	–	–	–
1‐Pentene, 3‐ethyl‐3methyl	3282	810.9	8.93	–	–	–
4‐Pentanoic acid, 2‐methoxy‐, methyl ester	3328	814.7	8.87	8.32	–	–
2‐Hexene, 4,4,5‐trimethyl‐	3365	817.7	8.59	–	–	–
1,5‐Heptadiene‐3,4‐diol, 2,5‐dimethyl	4037	872.6	6.53	–	–	–
Oxalic acid, cyclohexyl hexyl esther	3756	849.6	6.21	5.35	–	10.98
2‐Pentene, 4,4‐dimethyl	3799	853.1	5.69	14.47	–	–
1‐Butene, 2,3,3‐trimethyl‐	3878	859.6	5.27	5.25	–	4.37
1,5‐Heptadien‐4‐one, 3,3,6‐trimethyl‐	3521	830.4	4.46	4.34	–	–
1‐Propene, 3,3‐diethoxy‐	3353	816.7	–	8.24	–	–
2,3,3‐Trimethyl‐, 1‐hexene	3398	820.4	–	8.27	–	–
2,6‐ Octadiene, 2,4‐dimethyl‐	4071	875.3	–	6.72	–	–
3,5‐ Hexadien‐2‐ol	2933	NA	–	–	6.77	–
Cyclopropanecarboxylic acid, cyclohexylmethyl ester	3379	818.9	–	–	2.90	–
2‐Hexen‐1‐ol, 2‐ethyl‐	3523	830.6	–	–	2.01	–
3‐Methyl‐2‐butanoic acid, 4‐methylpenthyl ester	3847	857.1	–	–	2.63	–
Cyclohexanemethanol	4042	873.0	–	–	8.68	7.57
2,4‐Pentadien‐1‐ol, 3‐propyl‐, (2Z)‐	4583	915.9	–	–	2.28	–
3,9‐Epoxytricyclo(4,2,1,1(2,5)dec‐7‐en‐10‐ol, 9,10‐ dimethyl‐	9719	1297.3	–	–	5.14	–
Cyclopropanecarboxylic acid, 2‐[[2‐[[2‐[(2‐pentylcyclopropylmethyl)cyclopropyl]methyl]cyclopropyl]methyl	15 460	1802.1	–	–	4.16	–
1,7‐Octadiene, 2,3,3‐trimethyl‐	3372	818.3	–	–	–	4.80
Hexanoic acid	4328	896.3	–	–	–	4.44
4‐Methyl‐2,3‐hexadien‐1‐ol	2932	NA	–	–	–	4.04
2‐Octanol, 8,8‐dimethoxy‐2,6‐dimethyl‐	3332	815.0	–	–	–	2.86
Oxirane, 2‐methyl‐3‐propyl‐, cis‐	2373	NA	–	–	–	2.25
Homosalate	15 462	1802.3	–	–	–	2.07

*Note*: Compound identifications are based on spectral matching (≥80%) against the NIST 17 library and should be interpreted as provisional annotations, not definitive structural identifications.

By contrast, *T. tipu* leachates followed a markedly different chemical trajectory. Cyclohexanemethanol (8.68%) and 3,5‐hexadien‐2‐ol (6.77%) were prominent at D7, whereas oxalic acid (cyclohexyl hexyl ester) sharply increased to 10.98% at D14, with cyclohexanemethanol persisting at 7.57%. This pattern indicates a selective retention of oxygenated aliphatics and a progressive enrichment of low‐molecular‐weight organic acids over time.

Spectrophotometric assays corroborated the chromatographic divergence between species (Table 2). In *H. heptaphyllus*, total polyphenol concentrations declined markedly from 565 ± 49 mg L^−1^ at D7 to 291 ± 24 mg L^−1^ at D14. By contrast, *Tipuana tipu* exhibited a modest increase over the same period, rising from 542 ± 12 to 604 ± 62 mg L^−1^. The interspecific contrast was even more pronounced in condensed tannin content: *T. tipu* maintained concentrations approximately three‐fold higher than *H. heptaphyllus* at both time points (D7: 373 ± 15 *versus* 131 ± 14 mg L^−1^; D14: 402 ± 21 *versus* 128 ± 23 mg L^−1^).

**Table 2:** Total polyphenols and tannins analysis of *H. heptaphyllus* and *T. tipu*

Sample	Total polyphenols (mg L^−1^)	Tannins (mg L^−1^)
*H. heptaphyllus* (D7)	565.5 ± 49.15	131.3 ± 14.11
*H. heptaphyllus* (D14)	290.6 ± 23.69	127.6 ± 23.18
*T. tipu* (D7)	542.0 ± 11.51	373.1 ± 15.00
*T. tipu* (D14)	603.5 ± 61.55	402.1 ± 21.36

### 3.2 Mosquito's life‐history traits

**Figure 2:**
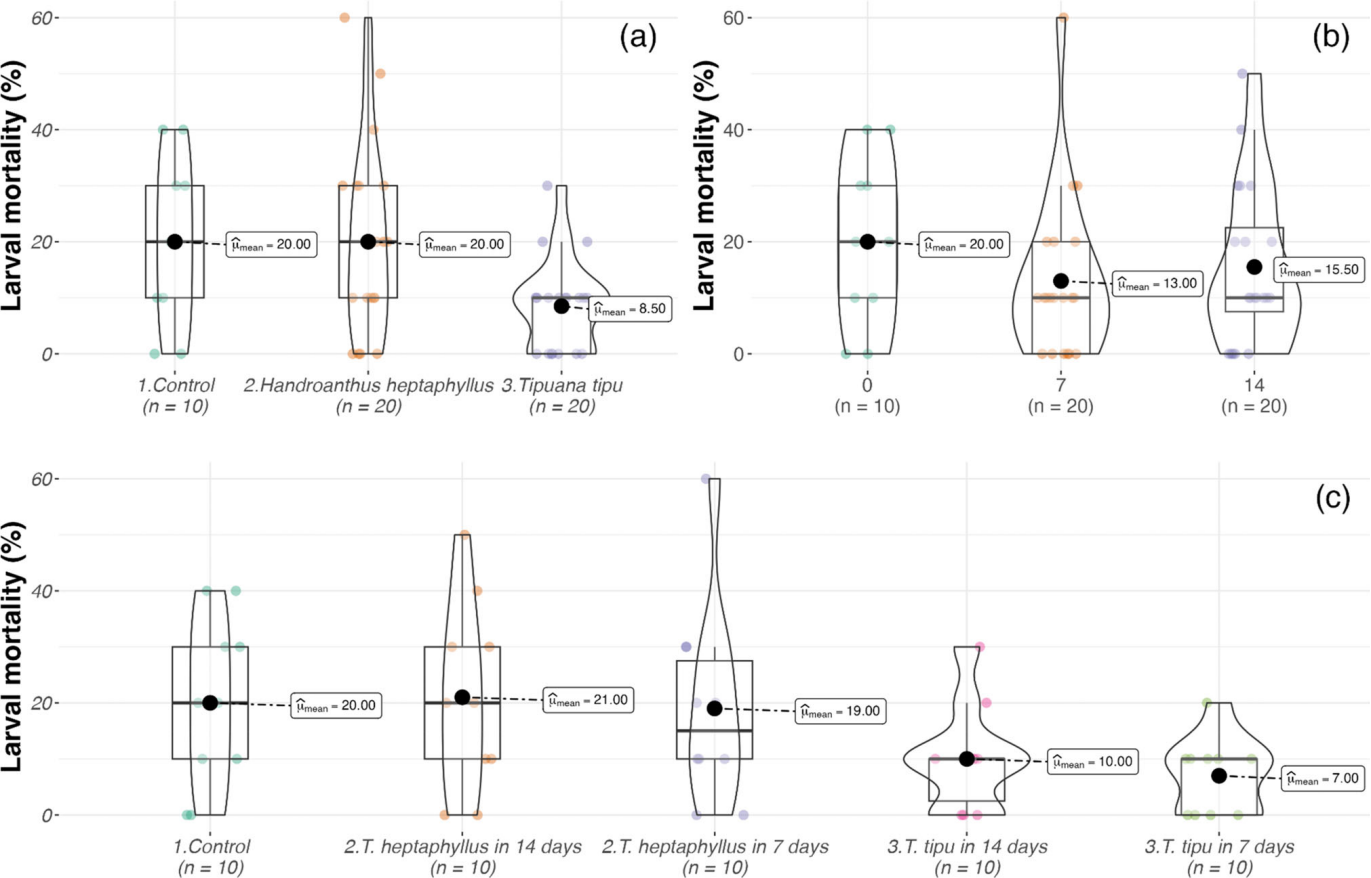
Violin and box plots showing larval mortality (%) of *A. aegypti* in Set A as a function of (a) litter species, (b) leaching duration and (c) their interaction. Boxes, interquartile range; horizontal bars, medians; black circles, means.

Larval survival exhibited species‐specific responses to leaf‐litter chemistry. In the 25% leachate screen (Set A), mortality was influenced solely by litter species [GLMM, χ^2^ = 19.4, *P* < 0.001; Table 3(a)]. Mortality in control microcosms averaged 20 ± 15%, statistically indistinguishable from the D7 and D14 leachates of *H. heptaphyllus* (19 ± 18% and 21 ± 17%, respectively). In sharp contrast, 25% *T. tipu* leachates reduced mortality to 7 ± 7% at D7 and to near‐zero levels by D14, suggesting a net nutritive effect at low concentrations (Fig. 2).

**Table 3:** Two‐way factorial GLMM performed to compare the isolated effect of litter species and leaching time, and their interactive effects on life‐history traits of *A. aegypti* mosquito cohorts.

GLMM	npar	AIC	BIC	LogLik	Deviance	Chisq	Df	Pr(>Chisq)	Contrast analysis
a. Mortality (%)									
Null	4	415.7	423.3	−203.8	407.7				
Leaf litter	7	413.2	426.6	−199.6	399.2	8.46	3	0.037	*T. tipu* < *H. heptaphyllus* = Control
Null	5	409.6	419.2	−199.8	399.6				
Leaching time	7	413.2	426.6	−199.6	399.2	0.38	2	0.828	
Null	3	414.0	419.7	−204.0	408.0				
Interaction	7	413.2	426.6	−199.6	399.2	8.79	4	0.066	
b. Days to emerge									
Null	5	585.2	600.1	−287.6	575.2				
Leaf litter	8	588.1	611.9	−286.1	572.1	3.10	3	0.3769	
Null	6	587.2	605.1	−287.6	575.2				
Leaching time	8	588.1	611.9	−286.1	572.1	3.13	2	0.2095	
Null	4	599.1	610.9	−295.5	591.1				
Interaction	8	588.1	611.9	−286.1	572.1	18.96	4	<0.001	*H. heptaphyllus* (D14) < *T. tipu* (D7) = *H. heptaphyllus* (D7) = *T. tipu* (D14) < Control
c. Adult size (mm)									
Null	5	165.4	185.6	−77.7	155.4				
Leaf litter	8	154.4	186.7	−69.2	138.4	17.05	3	<0.001	Control < *H. heptaphyllus* < *T. tipu*
Null	6	152.1	176.4	−70.1	140.1				
Leaching time	8	154.4	186.7	−69.2	138.4	1.77	2	0.412	
Null	4	188.2	204.4	−90.1	180.2				
Interaction	8	154.4	186.7	−69.2	138.4	41.85	4	<0.001	Control < *H. heptaphyllus* (D7 < D14) < *T. tipu (14 < 7)*
d. Asymmetry (mm)									
Null	5	−562.9	−542.7	286.5	−572.9				
Leaf litter	8	−558.1	−525.8	287.0	−574.1	1.16	3	0.762	
Null	6	−556.3	−532.0	284.1	−568.3				
Leaching time	8	−558.1	−525.8	287.0	−574.1	5.81	2	0.055	
Null	4	−555.6	−539.5	281.8	−563.6				
Interaction	8	−558.1	−525.8	287.0	−574.1	10.46	4	<0.001	Control = *T. tipu* (D7) = *H. heptaphyllus* (D7) < *T. tipu* (D14) = *H. heptaphyllus* (D14)

*Note*: Larval mortality rate (a) considered replicates as a random effect, and days to emerge (b), adult size (c) and body asymmetry (d) considered sex and replicates as random effects. Additionally, contrast analyses to compare response variables for significant explanatory variables.

**Figure 3:**
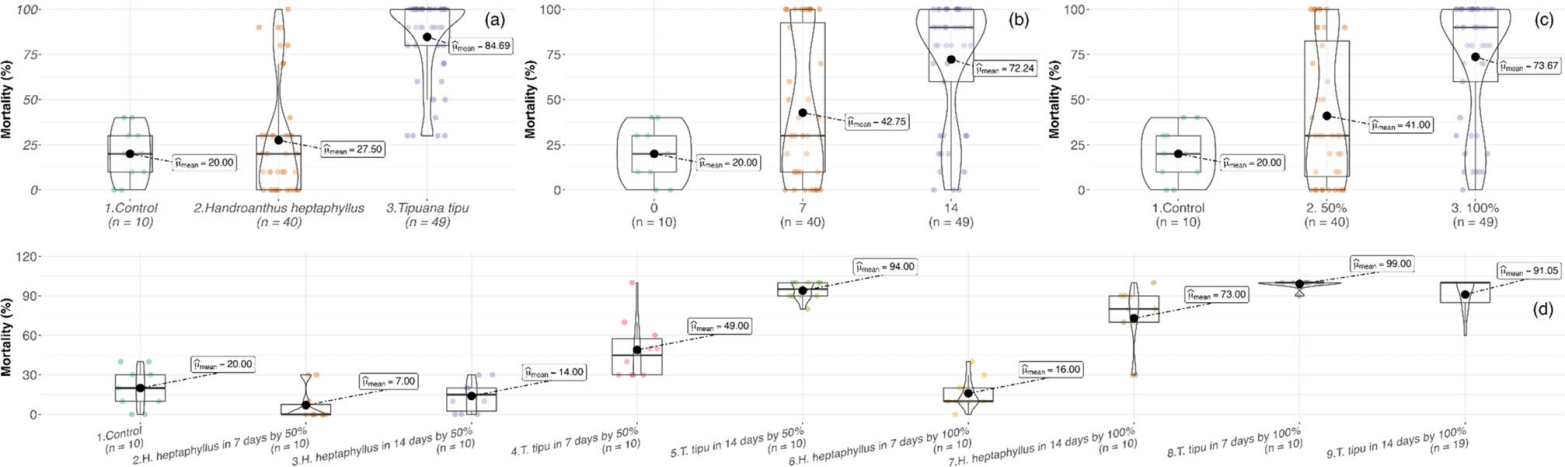
Violin and box plots of *A. aegypti* larval mortality (%) in Set B across (a) litter species, (b) leaching duration, (c) leachate concentration and (d) their interaction. Boxes, interquartile range; horizontal bars, medians; black circles, means.

When leachate concentration was varied (Set B), larval mortality was jointly governed by species identity, leaching duration, leachate strength, and their interactions [three‐way GLMM, all *P* < 0.01; Table 4(a)]. Full‐strength (100%) *T. tipu* extracts were highly toxic, inducing 99 ± 3% mortality at D7 of leaching and 92 ± 13% at D14. Even at 50% strength, *T. tipu* remained lethal, with 94 ± 7% mortality. By contrast, *H. heptaphyllus* leachates produced ≤16 ± 12% mortality across all concentration–time combinations, values statistically indistinct from the control (Fig. 3).

**Table 4:** Three‐way factorial GLMM performed to compare the isolated effect of litter species, leaching time, leaching concentration and their interactive effects on life‐history traits of *A. aegypti* mosquito cohorts.

GLMM	npar	AIC	BIC	LogLik	Deviance	Chisq	Df	Pr (>Chisq)
a. Mortality (%)								
Null model	7	64.461	82.627	−25.230	50.461			
Litter Species	11	−87.909	−59.363	54.955	−109.909	160.37	4	<0.001
Null model	7	−8.613	9.553	11.306	−22.613			
Leaching Time	11	−87.909	−59.363	54.955	−109.909	87.296	4	<0.001
Null model	7	−1.696	16.470	7.848	−15.696			
Leaching Concentration	11	−87.909	−59.363	54.955	−109.909	94.213	4	<0.001
Null model	5	75.296	88.271	−32.648	65.296			
Litter Species: Leaching Time	11	−87.909	−59.363	54.955	−109.909	175.21	6	<0.001
Null model	4	77.242	87.622	−34.621	69.242			
Litter Species: Leaching Concentration	11	−87.909	−59.363	54.955	−109.909	179.15	7	<0.001
Null model	5	19.140	32.115	−4.570	9.14			
Leaching Time: Leaching Concentration	11	−87.909	−59.363	54.955	−109.91	119.05	6	<0.001
Null model	3	99.650	107.435	−46.825	93.65			
Litter Species: Leaching Time: Leaching Conc.	11	−87.909	−59.363	54.955	−109.91	203.56	8	<0.001
b. Days to emerge								
Null model	7	910.79	933.63	−448.40	896.79			
Litter Species	11	907.97	943.86	−442.98	885.97	10.821	4	0.028
Null model	7	907.03	929.87	−446.51	893.03			
Leaching Time	11	907.97	943.86	−442.98	885.97	7.060	4	0.133
Null model	7	902.61	925.45	−444.30	888.61			
Leaching Concentration	11	907.97	943.86	−442.98	885.97	2.639	4	0.619
Null model	5	916.75	933.06	−453.37	906.75			
Litter Species: Leaching Time	11	907.97	943.86	−442.98	885.97	20.776	6	0.002
Null model	4	909.58	922.63	−450.79	901.58			
Litter Species: Leaching Concentration	11	907.97	943.86	−442.98	885.97	15.611	7	0.029
Null model	5	903.05	919.37	−446.53	893.05			
Leaching Time: Leaching Concentration	11	907.97	943.86	−442.98	885.97	7.084	6	0.313
Null model	3	920.28	930.07	−457.14	914.28			
Litter Species: Leaching Time: Leaching Conc.	11	907.97	943.86	−442.98	885.97	28.314	8	< 0.001
c. Adult size								
Null model	8	−1425.8	−1393.4	720.90	−1441.8			
Litter Species	12	−1426.3	−1377.7	725.15	−1450.3	8.490	4	0.075
Null model	8	−1423.4	−1391.0	719.69	−1439.4			
Leaching Time	12	−1426.3	−1377.7	725.15	−1450.3	10.921	4	0.027
Null model	8	−1420.2	−1387.9	718.13	−1436.2			
Leaching Concentration	12	−1426.3	−1377.7	725.15	−1450.3	14.037	4	0.007
Null model	6	−1416.3	−1392.0	714.16	−1428.3			
Litter Species: Leaching Time	12	−1426.3	−1377.7	725.15	−1450.3	21.974	6	0.001
Null model	5	−1416.8	−1396.5	713.39	−1426.8			
Litter Species: Leaching Concentration	12	−1426.3	−1377.7	725.15	−1450.3	23.51	7	0.001
Null model	6	−1424.0	−1399.7	717.98	−1436.0			
Leaching Time: Leaching Concentration	12	−1426.3	−1377.7	725.15	−1450.3	14.336	6	0.026
Null model	4	−1418.8	−1402.6	713.39	−1426.8			
Litter Species: Leaching Time: Leaching Conc.	12	−1426.3	−1377.7	725.15	−1450.3	23.517	8	0.002
**d. Asymmetry**								
Null model	8	−424.19	−397.12	220.10	−440.19			
Litter Species	11	−420.14	−382.91	221.07	−442.14	1.9462	3	0.583
Null model	8	−422.68	−395.60	219.34	−438.68			
Leaching Time	11	−420.14	−382.91	221.07	−442.14	3.4615	3	0.326
Null model	8	−417.94	−390.87	216.97	−433.94			
Leaching Concentration	11	−420.14	−382.91	221.07	−442.14	8.1956	3	0.042
Null model	6	−426.54	−406.23	219.27	−438.54			
Litter Species: Leaching Time	11	−420.14	−382.91	221.07	−442.14	3.599	5	0.608
Null model	5	−420.20	−403.28	215.10	−430.20			
Litter Species: Leaching Concentration	11	−420.14	−382.91	221.07	−442.14	11.934	6	0.063
Null model	6	−419.76	−399.46	215.88	−431.76			
Leaching Time: Leaching Concentration	11	−420.14	−382.91	221.07	−442.14	10.374	5	0.065
Null model	4	−421.74	−408.20	214.87	−429.74			
Litter Species: Leaching Time: Leaching Conc.	11	−420.14	−382.91	221.07	−442.14	12.399	7	0.088

*Note*: Larval mortality rate (a) considered replicates as a random effect, and days to emerge (b), adult size (c) and body asymmetry (d) considered sex and replicates as random effects.

**Figure 4:**
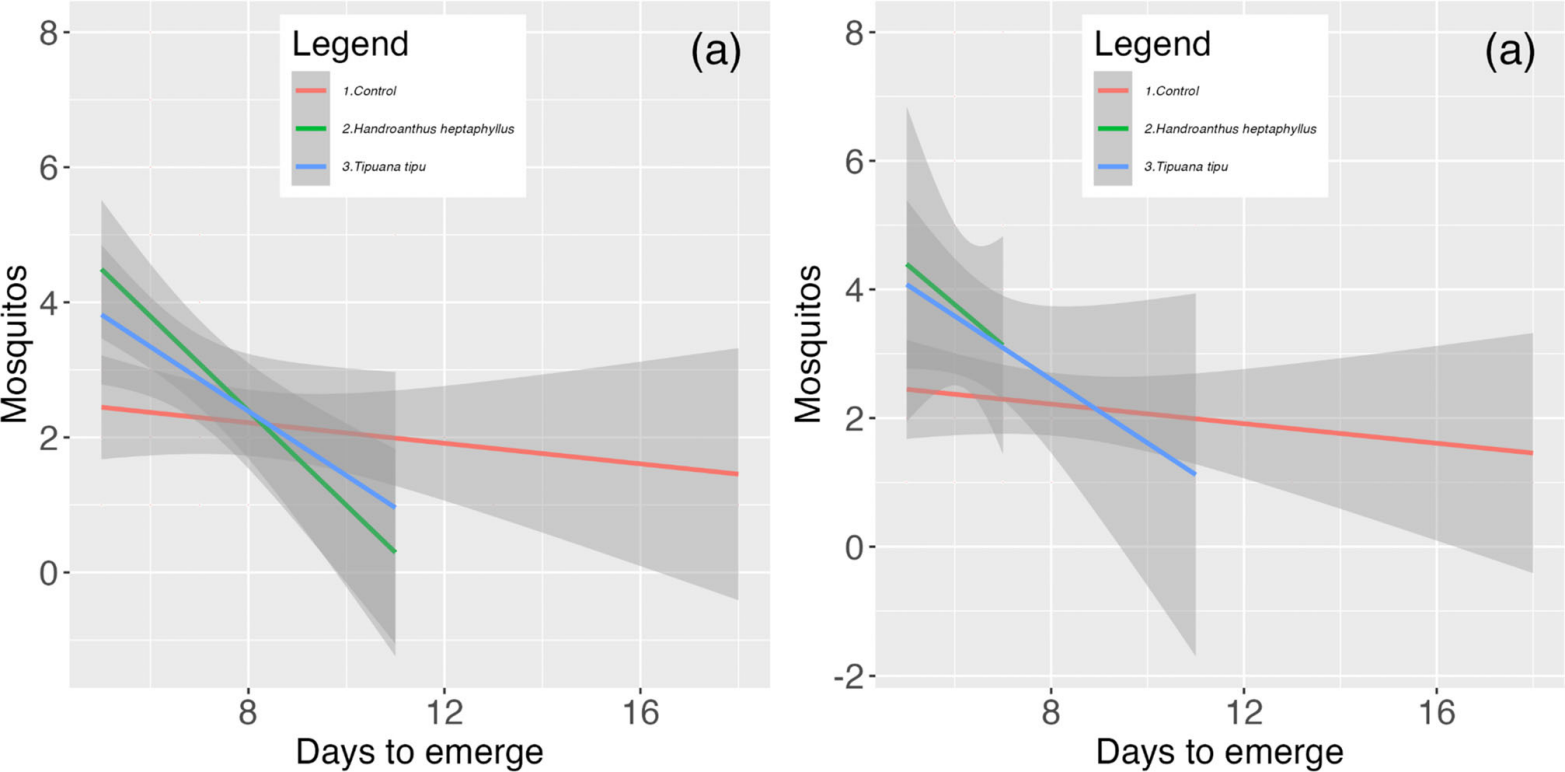
Development time (days to adult emergence) of *A. aegypti* across litter species treatments (Control, *H. heptaphyllus* and *T. tipu*) after (a) 7and (b) 14 days of leaf leaching in Set A.

**Figure 5:**
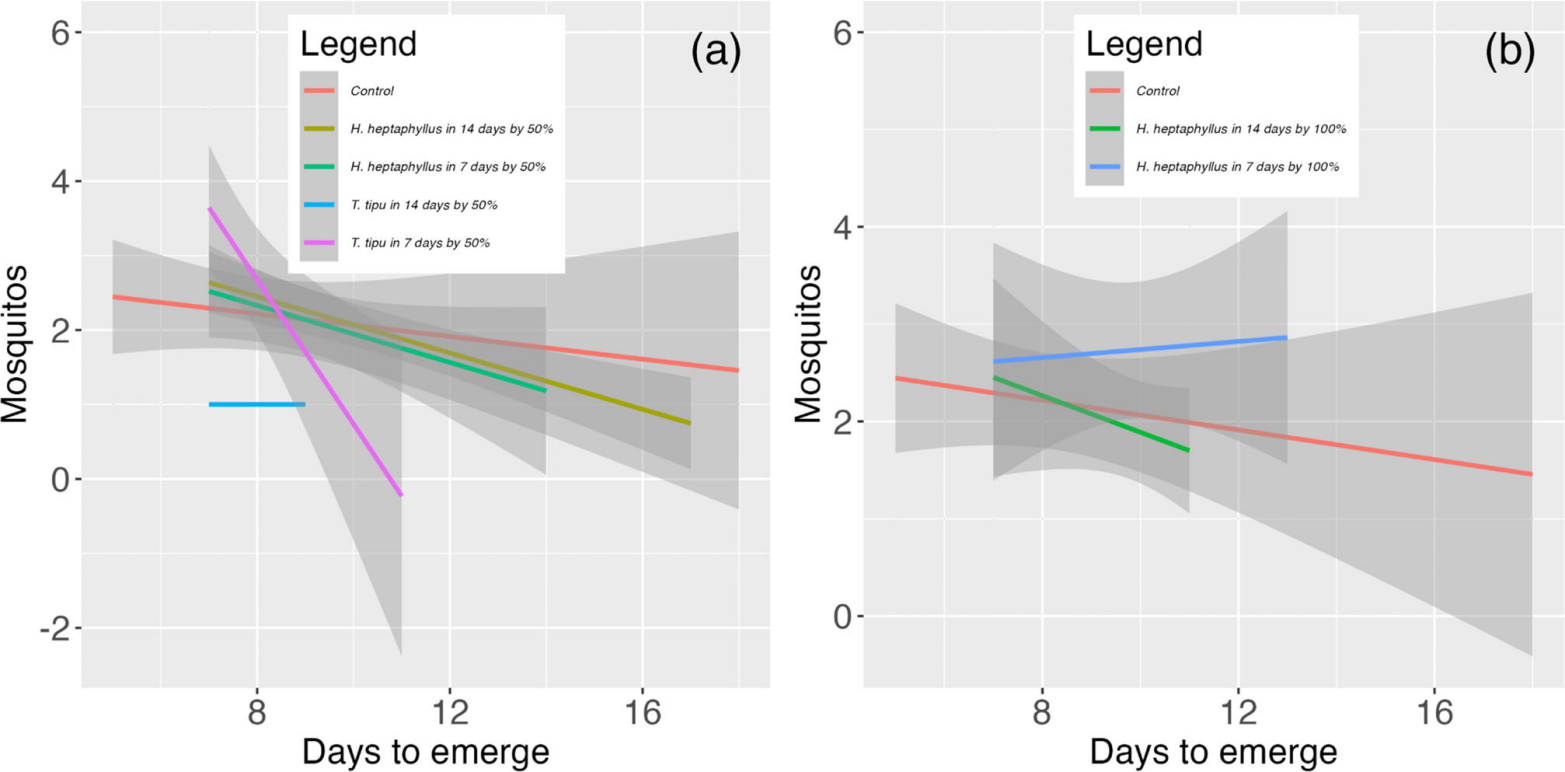
Development time (days to adult emergence) of *A. aegypti* across litter species treatments (Control, *H. heptaphyllus* and *T. tipu*) in Set B, following 7 and 14 days of leaf leaching at (a) 50% and (b) 100% leachate concentration.

Developmental timing closely paralleled survival outcomes. In Set A, mean time to adult emergence differed significantly only for the litter species × leaching time interaction [Table 3(b)]. The D14 leachate of *H. heptaphyllus* accelerated emergence to 6.27 ± 0.70 days, nearly 2 days earlier than the control (8.22 ± 2.67 days), whereas both *T. tipu* treatments yielded intermediate development times (~6.7 days; Fig. 4). In Set B, emergence timing was governed by a significant three‐way interaction among species, concentration and leaching duration [Table 4(b)]. The slowest cohorts (~10.2 days) occurred in 50% and 100% D14 *H. heptaphyllus* leachates, whereas the fastest (~7.8 days) developed in 50% *T. tipu* leachate (Fig. 5).

**Figure 6:**
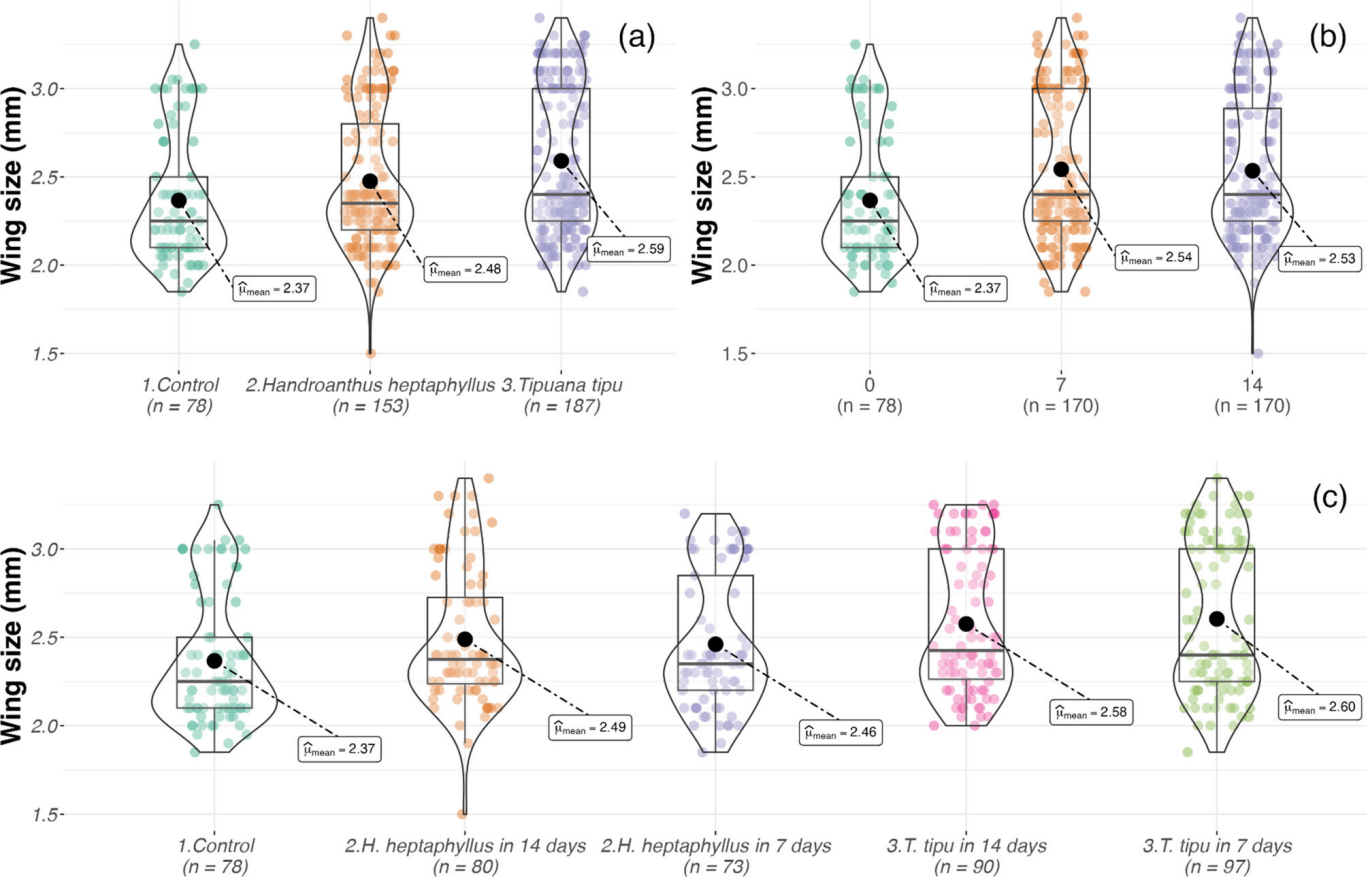
Violin and box plots of *A. aegypti* adult wing length (mm) in Set A across (a) litter species, (b) leaching duration and (c) their interaction. Boxes, interquartile range; horizontal bars, medians; black circles, means.

**Figure 7:**
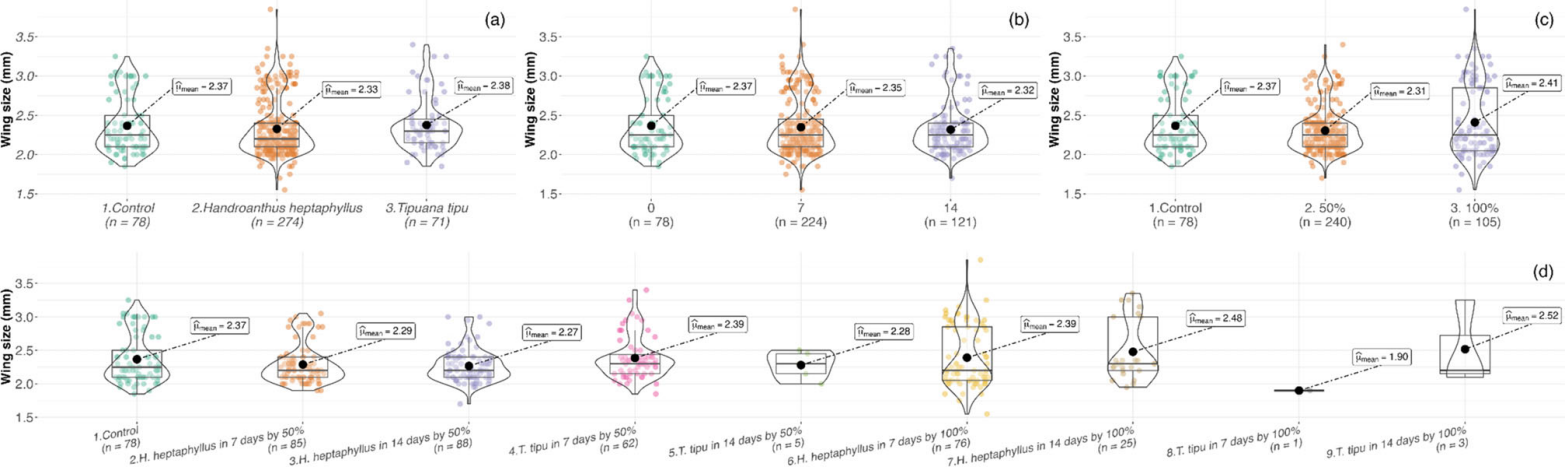
Violin and box plots of *A. aegypti* adult wing length (mm) in Set B across (a) litter species, (b) leaching duration, (c) leachate concentration and (d) their interaction. Boxes, interquartile range; horizontal bars, medians; black circles, means.

Adult body size exhibited nonlinear responses across the same treatment factors. In Set A (25% leachates), wing length varied by litter species (GLMM, χ^2^ = 16.1, *P* < 0.001) and its interactions [Table 3(c)]. Control mosquitoes were the smallest (2.36 ± 0.09 mm), those reared in *H. heptaphyllus* were slightly larger (~2.47 mm), and *T. tipu* produced the largest individuals (2.57–2.60 mm; Fig. 6). In Set B, adult size was influenced by leachate concentration, leaching duration and all interaction terms [Table 4(c)]. The largest adults (2.73 ± 0.07 mm) emerged from 100% D14 *T. tipu* leachate, whereas the smallest (~2.15 mm) developed in 100% D7 *T. tipu* leachate and in the most concentrated *H. heptaphyllus* treatments (Fig. 7).

**Figure 8:**
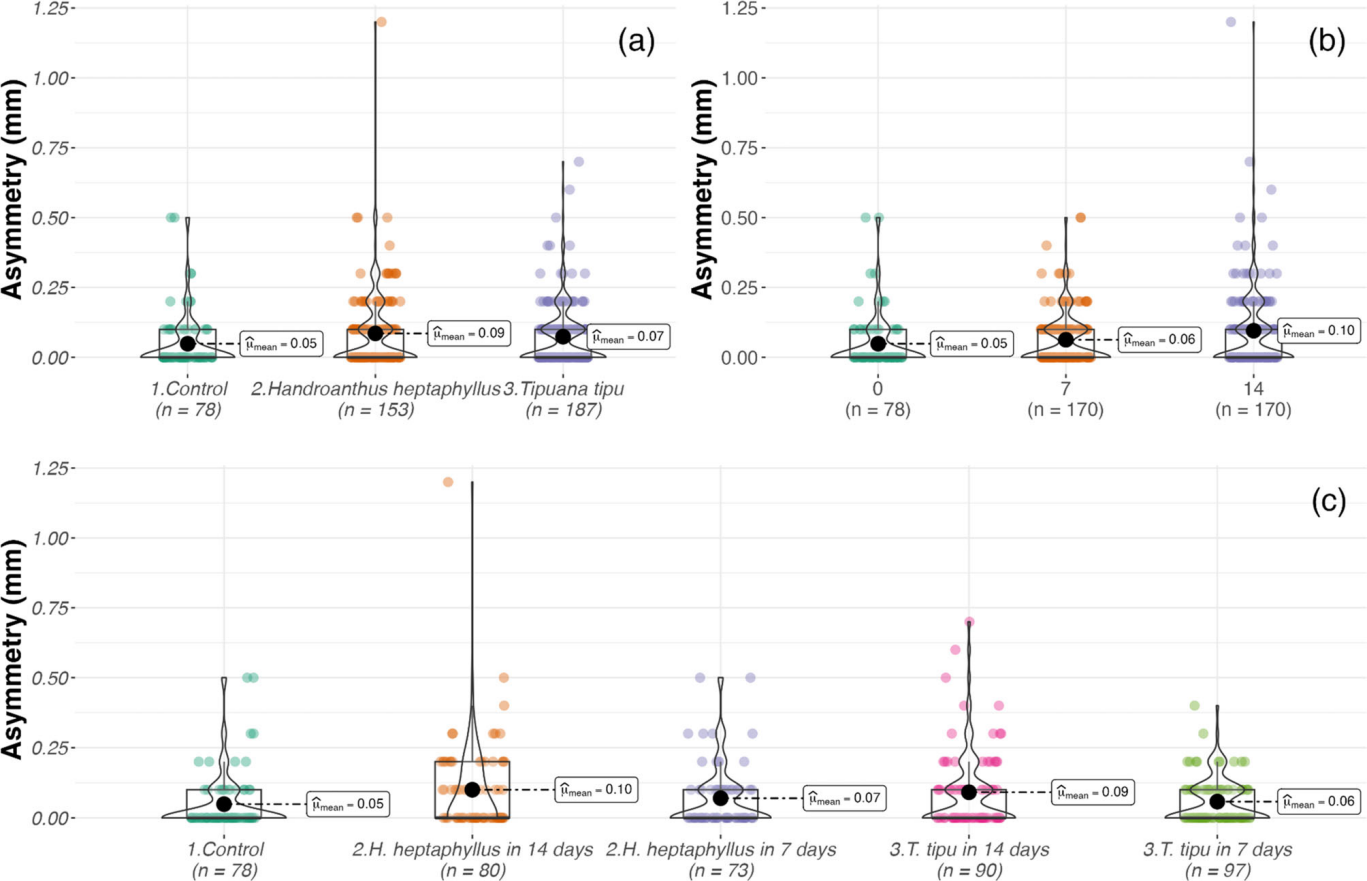
Violin and box plots of *A. aegypti* wing asymmetry (mm) in Set A across (a) litter species, (b) leaching duration and (c) their interaction. Boxes, interquartile range; horizontal bars, medians; black circles, means.

**Figure 9:**
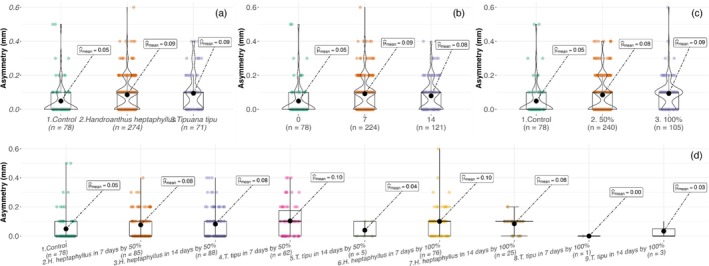
Violin and box plots of *A. aegypti* wing asymmetry (mm) in Set B across (a) litter species, (b) leaching duration, (c) leachate concentration and (d) their interaction.

Fluctuating wing asymmetry (FA) remained low overall but was modulated by treatment interactions. In Set A, only the species × leaching time interaction was significant [Table 3(d)], with FA increasing from 0.05 ± 0.10 mm in controls to 0.09–0.10 mm in D14 leachates, particularly in *H. heptaphyllus* (Fig. 8). In Set B, FA increased with leachate concentration (GLMM, χ^2^ = 7.4, *P* = 0.006) and was further shaped by higher‐order interactions, peaking in 100% *H. heptaphyllus* (0.12 ± 0.13 mm) and 50% D7 *T. tipu* (0.10 ± 0.11 mm; Table 4(d), Fig. 9). Controls consistently exhibited the lowest asymmetry (0.05 ± 0.10 mm).

## 4 DISCUSSION

### 4.1 Key ecological outcomes

Our findings indicate that foliar leachates from two dominant urban tree species may act as ecological filters for *A. aegypti*, influencing larval survival and adult phenotype in a species‐, concentration‐, and time‐dependent manner. High mortality occurred exclusively under the 50% and 100% *T. tipu* treatments, for both D7 and D14 leachates (92–99%; Set B). No other concentration–age combination produced such levels of lethality. Importantly, 25% *T. tipu* did not induce mortality; instead, it consistently supported higher survival relative to the control. Conversely, *H. heptaphyllus* leachates remained nonlethal across all treatments, with mortality never >16%. These contrasting biological responses, spanning six‐fold differences in survival, 2‐day shifts in developmental timing, 25% variation in body size, and elevated wing asymmetry, aligned with species‐specific chemical trajectories, with a temporally persistent profile enriched in tannins and oxalate‐related metabolites in *T. tipu versus* a rapid decline in phenolics and the prominence of alkenes in *H. heptaphyllus*.

Taken together, these results suggest that a single tree species may transition functionally from ‘fertilizer’ to ‘insecticide’ depending on shifts in leachate chemistry, pointing to a self‐renewing, landscape‐embedded process that could contribute to vector suppression alongside existing management strategies.^9^, ^19^, ^28^ By integrating untargeted metabolomic profiling with factorial bioassays, this study may offer a conceptual framework for exploring how urban‐greening decisions may interact with mosquito ecology and highlights urban leaf litter as a previously under‐recognized driver in the environmental dynamics of arboviral disease transmission.

### 4.2 Species‐specific chemistry frames ecological outcomes

Our chromatographic analyses revealed that leachates from *T. tipu* and *H. heptaphyllus* follow markedly divergent chemical trajectories over time. In *H. heptaphyllus*, total phenolics and condensed tannins declined by ≈50% between D7 and D14, accompanied by a compositional shift from short‐chain alkenes to oxidized esters, a pattern consistent with the early loss of water‐soluble defense metabolites commonly reported for hardwood litter.^20^, ^24^, ^29^, ^30^ By contrast, *T. tipu* retained a comparatively stable profile dominated by oxygenated aliphatics and showed a three‐fold increase in condensed tannins together with the accumulation of an oxalate‐related ester (oxalic acid, cyclohexyl hexyl ester). This pattern may align with the behavior of leguminous foliage enriched in hydrolyzable tannins, which often leach gradually and exhibit slower microbial degradation.^21^, ^23^, ^31^ These species‐specific chemical fingerprints are consistent with the divergent larval outcomes observed in our bioassays and suggest plausible mechanistic pathways that merit further investigation, while recognizing that the processes involved were not directly quantified in our study.^31^, ^32^

### 4.3 Dose–response logic of mosquito's life‐history traits

Larval survival closely tracked a composite gradient of condensed tannins, oxalic acid (cyclohexyl hexyl ester), and dissolved organic carbon (DOC). The observed increase in larval survival at 25% *T. tipu* leachate may reflect a synergistic interaction between the baseline *Spirulina* diet and leachate‐derived DOC, which together could stimulate microbial biomass and thereby enhance the availability of microbially mediated food resources, rather than indicating that the leachate itself functions as a direct nutrient source.^33^ Accordingly, this pattern is more cautiously interpreted as an emergent outcome of the combined dynamics of *Spirulina* supplementation, DOC enrichment and microbiota proliferation, consistent with previous studies suggesting that microbial growth can modulate positive nutritional effects in container‐breeding mosquito systems.^34^ Also, although the oxalate detected corresponds to an esterified derivative rather than free oxalate, its presence may still indicate oxalate‐related chemistry that could influence larval physiology in indirect ways, with low levels of oxalate conferring antioxidant protection during ecdysis^35^, ^36^.

When leachate concentration reached ≥50% or when aging extended to 14 days, mortality surpassed 90%. These high mortality levels are consistent with hypotheses proposed in other systems, such as potential tannin‐protein interactions^37^ or precipitation phenomena^38^ associated with oxalate‐related compounds, both of which have been reported to affect midgut stability and epithelial integrity in various taxa^39^. Although our study did not directly measure these processes, they offer plausible explanations that align with the observed patterns. By contrast, *H. heptaphyllus* leachates produced mortality levels similar to the control across all treatments, which is compatible with their lower tannin concentrations and the rapid decline of phenolic compounds during leachate aging. The predominance of unsaturated alkenes in *H. heptaphyllus*, generally characterized by low larvicidal activity at environmentally relevant concentrations, further corresponds with the limited toxicity observed.^40^, ^41^

Both leachate types accelerated development relative to the control, yet the most rapid emergence (~6 days) was observed in D14 *H. heptaphyllus* leachates, despite their low energetic content. In insects, accelerated metamorphosis under sublethal chemical exposure has been documented as a potential compensatory strategy,^9^, ^15^ often reflecting a trade‐off between growth and survival.^5^, ^19^, ^42^ In the present context, the temporal decline in phenolic compounds in *H. heptaphyllus* leachates may have reduced chemical stress sufficiently to allow for quicker molting,^43^ whereas residual DOC could have supported microbial proliferation that partially mitigated nutritional limitations.^33^ Conversely, the slowest development (~10 days) occurred in full‐strength *H. heptaphyllus* leachate, a pattern that may reflect a combination of restricted energy availability and mild chemical stress delaying metamorphosis^9^, ^19^, consistent with broader conceptual models of stress‐associated developmental postponement^35^, ^41^.

Wing length, a key proxy for adult mosquito fitness^44^, suggests that adult body size exhibited a pronounced, nonlinear response to *T. tipu* concentration and aging. The largest adults emerged from 25% *T. tipu* in Set A, a pattern that may be consistent with a transient enhancement in resource availability potentially linked to labile DOC and other readily metabolizable compounds such as cyclohexanemethanol, which can support microbial processing and thereby increase the quality of larval food resources^34^, ^45^. However, the smallest adults occurred in the D14 100% *T. tipu* leachate (~2.15 mm), a pattern consistent with strong chemical stress at high concentrations. At higher concentrations, elevated tannin and oxalate‐related compounds may have counteracted these benefits, coinciding with pronounced reductions in adult body size.^46^, ^47^ *H. heptaphyllus* displayed an inverse pattern, with its lower tannin content may reduce toxic stress yet also limit energetic inputs.^46^, ^47^ resulting in intermediate‐sized adults across treatments. These shifts in body size have epidemiological implications^44^, as larger *Aedes* females are generally more fecund, longer‐lived and may exhibit higher vector competence^47^, ^48^, ^49^. These results clarify that 100% *T. tipu* leachates, regardless of time, do not enhance adult size; instead, they markedly reduce it. Accordingly, low‐dose *T. tipu* leachates could, under certain conditions, favor traits associated with increased vectorial capacity, whereas more concentrated leachates appear to suppress adult performance.^47^, ^48^, ^49^

Bilateral wing asymmetry (FA) remained below 0.05 mm in control groups but increased to 0.10–0.12 mm under the most chemically stressful treatments, values that fall within ranges commonly associated with developmental instability in mosquitoes^9^, ^49^. The highest FA levels occurred in 100% *H. heptaphyllus* (D7) and 50% *T. tipu* (D7), conditions in which chemical stress may have been sufficient to perturb morphogenesis without causing acute lethality. Elevated FA has been linked in previous studies to reduced flight performance and diminished host‐seeking efficiency^9^, ^12^, ^42^, suggesting that even individuals with otherwise normal body size may experience compromised postemergence survival or vectorial potential under such conditions.

### 4.4 Implications for urban vector management

Our findings suggest that the ecological role of urban leaf litter is influenced by both botanical identity and hydrological conditions.^19^, ^31^ *T. tipu*, a dominant species in Chapecó's street canopy, appears to function as a chemical ‘double‐edged sword’: at low dilution, runoff may support *A. aegypti* performance, whereas more concentrated leachates, such as those potentially formed after prolonged dry periods followed by intense rainfall, were associated with substantially higher larval mortality in our assays. By contrast, *H. heptaphyllus* leaf litter produced comparatively neutral effects, eliciting mild physiological stress but no consistent lethal responses. These functional differences indicate that tree species composition could, in principle, influence mosquito developmental environments in urban settings. While caution is warranted in translating experimental findings to management actions, incorporating knowledge of plant–vector interactions into urban landscape planning may provide an additional, ecologically grounded layer to integrated vector management strategies^9^, ^19^.

## 5 CONCLUSIONS

Our factorial bioassays, combined with detailed metabolite profiling, suggest that urban leaf litter may not act merely as a passive substrate, but instead, rather as a chemically dynamic matrix capable of nourishing, stressing or reducing the performance of *A. aegypti* depending on botanical origin, leachate concentration and decomposition stage. At low dilution (25%), *T. tipu* leachates enhance survival, accelerate development and produce larger adults; however, at higher concentrations (50–100%) and both leaching durations (D7 and D14), *T. tipu* consistently produces >90% mortality and substantially smaller adults. By contrast, *H. heptaphyllus* remains chemically benign across the same conditions, generating low mortality and intermediate adult sizes. These clarified patterns reinforce that *T. tipu* transitions from ‘fertilizer‐like’ to ‘insecticide‐like’ effects only when concentrations exceed 25% and not merely at D14. It raises the possibility that vector pressure could be influenced, at least in part, through the management of litter inputs already embedded within the urban canopy. From this perspective, ecologically informed design choices may complement existing vector control programs, which generally rely on externally applied chemical interventions.

We acknowledge several limitations. The microcosm design simplifies hydrological variation and excludes ecological interactions, such as predation and competition.^6^, ^42^, ^50^ Additionally, potential nontarget effects were inferred rather than measured directly. Future studies should incorporate field‐based litterfall monitoring, rainfall dynamics and broader aquatic community assessments to evaluate whether these patterns hold under realistic urban conditions. Targeted isolation and dose–response assays of candidate metabolites also will be essential for clarifying causal mechanisms. Even so, our results refine prevailing models of container‐mosquito ecology by suggesting that leaf litter may function not simply as inert detritus, but instead as a chemically active filter whose ecological role may range from nutrient subsidy to larval suppression. Considering this dimension within urban forestry planning, for example, deploying *T. tipu* in areas susceptible to litter accumulation and favoring *H. heptaphyllus* where ecological risks should be minimized, could offer an integrative, self‐renewing approach to reducing arboviral transmission in rapidly urbanizing subtropical regions.

## Data Availability

The data that support the findings of this study are available from the corresponding author upon reasonable request.
